# 

**Published:** 1863-01

**Authors:** 


### INDEX TO VOLUME VI.

Anatomy of Arteries—Power, - - - - - - - 41
Am. Med. Assoc., - - - - - 137, 139, 214, 276 286
Army Practice, Glimpses of, - - - - - - 142
Absorption, Curiosity, - - - - - - - - 186
Anæsthesia, National Reward for, - - - - 236
Army Correspondence—Eaton, - - - - - - - 354
Anæmia, Idiopathic, - - - - - - - - 441
Army Surgeon, The, - - - - - - 460, 461
Attenuation of Cuticle, - - - - - - - 474
Asthma—Verat. Virid.—Winch, - - - - - - 511
Atlantic Ocean, Pressure, - - - - - - - 517
Army Laboratory, - - - - - - - - 522
Atmosphere, Life in, - - - - - - - - 572

Brackett, Dr. Chas., Notice of Death, - - - - - 138
Bromine—Goldsmith, - - - - - - - 145
Bunions, - - - - - - - - - - - 189
Bromide of Ammonium, - - - - - - - 204
Burns, - - - - - - - - - - - 220
Bismuth, Substitutes for, - - - - - - - 221
Bladder, Pain in Diseased, - - - - - - - 232
Bobus, - - - - - - - - - - 321
Botanic Druggists, - - - - - - - - 325
Bowman, Handbook of Chemistry, - - - - - 330
Bartholow, Manual of Instructions, - - - - - - 470
Blaney, Prof. J. V. Z., - - - - - - - 473
Bread, Crust of, - - - - - - - - 521
Blood, Morbid Conditions—Flint, - - - - - 530

Consumption in N. E.—Bowditch, - - - - - - - 41
Conservative Medicine, - - - - - - 65, 561
Cholesterine, - - - - - - - - - - 187

Cazeaux' Midwifery, - - - - - - - - 191, 326
Cerebro Sp. Mening., - - - - - 122, 193, 260, 344
Cannabis Indica, Chronic Diarrhœa, - - - - 224
Cider in Erysipelas, - - - - - - - 225
Canada Lancet, - - - - - - - - - - 225
Camp Diarrhœa, - - - - - - - - 230, 346, 429
Chemistry—Brande & Taylor, - - - - - - 237
Camp Fever, Diff. Diagnosis, - - - - - - - 238
Calomel in the Army—Edwards, - - - - - - 254
Calomel and Tart. Emetic in Army, - - - 355, 409
Cholera in Malabar, - - - - - - - - - 381
Chloroform in Labor, - - - - - - - - 386
Camps, An Enemy in, - - - - - - - - 397
Colored Population, Circular, - - - - - - 478
Clinical Lectures—Holmes, - - - - 498, 526, 548
Camp Dysentery, - - - - - - - - - 505
Cantharides, Strangury from, - - - - - - 569

Debility and Diarrhœa of Soldiers, - - - - - - 81
Diagnosis Malarial Diathesis, - - - - - - 83
Diphtheria, - - - - - - 91, 109, 195, 477
Dialysis, - - - - - - - - - - - 97
Dissection Wound—R**, - - - - - - - 147
Dropsy—Turkish Bath, - - - - - - - 222
Dyspepsia—Rennet Wine, - - - - - - 223
Diabetic Urine, - - - - - - - - - - 232
Dis. of Women and Children—Bedford, - - - - 327
Disinfectants, - - - - - - - - - - 358

Epidemics, Cause of, - - - - - - - - 80
Epidemic in North West—Allen, - - - - - - 122
Enchondroma, - - - - - - - - - - 187
Eye and Ear Infirmary, Chicago, Reports, - - - - 334
Exemptions from Conscription, - - - - - - 370
Editorial Change, - - - - - - - - 384, 471
Enteritis—Intest. Obstruct., - - - - - - - 514
Enterotomy, - - - - - - - - - - 569
Errata, - - - - - - - - - - - - 576

Forces of Liv. Organism, - - - - - - - 205
Food, Nitrogen of, - - - - - - - - - 215
Facial Neuralgia, - - - - - - - - - 217
Fractures, Shortening after, - - - - - - - 229
Force, Lecture—Tyndall, - - - - - - - 293
Fibrinous Coagula in Heart, - - - - - - 331, 428
Foreign Correspondence, - - - 348, 414, 417, 433
Fracture of Thigh, - - - - - - - - 3[illegible]9
Formulæ, Miscellaneous, - - - - - - - 381

Gums, Lancing in Dentition, - - - - - - - 79
Glycerine locally in Fevers, - - - - 188
Glycerole of Tar, - - - - - - - 190
Graduates of the College, - - - - - - 190
Gonorrhœal Rheumatism, - - - - - - - 219
Ghosts, - - - - - - - - - - - 225
Gonorrhœa, - - - - - - - - - - 231
Glycerine, Uses of, - - - - - - - - 333
"Gouging," Contin. Hist. of Case—Holmes, - - - 352

Hernia, Strangulated—Moore, - - - - - - 1
Hopps' Trial, - - - - - - - - - 38, 162
Hospital Steward's Manual, - - - - - - - 40
Hypodermic Injections, - - - - - - - - 71
Homœopathy. - - - - - - - - - - 141
Health, Its Friends and Its Foes—Mussey, - - - 188
Hair, Lotion for, - - - - - - - - 189
Hæmorrhoids, Operation, - - - - - - - 220
Hay Asthma, - - - - - - - - - 221
Hæmatoxylon Deodorizer, - - - - - - - 221
Humors of the Craft, - - - - - - - 322
Headland, Action of Medicines, - - - - - - 329
Hamilton, Fractures and Dislocations, - - - - 329
Hammond, Military Hygiene, - - - - - - 373
Hernia, Radical Cure, - - - - - - - - 379
Hammond, Surg. Gen., - - - - - - - - - 471
Hobbies, - - - - - - - - - 472, 474
Hepatitis, Suppurative, - - - - - - - - - 473

Illinois State Med. Soc., - - - - - - - 93
Insanity and Intemperance—McFarland, - - - - 162, 183
Iodurated Frictions in Pleurisy, etc., - - - - - 190
Insanity, Phys. Disease in, - - - - - - - 211
Ice Cream, Topically, - - - - - - - - 223
Inoculation in Intermittent, - - - - - - - 226
Illegitimate Births, - - - - - - - - 232
Injections, Intra Uterine, - - - - - 287, 382
Iodine; Its Actions, etc., - - - - - - - 303
Insanity, Noisy and Restless, - - - - - - - 431
Introductory Address—Ingals, - - - - - - 481
Income Tax, Physicians, - - - - - - - 525

Joint Diseases, Treatment, - - - - - - - 209
Joint Hip, Functions of Round Ligament, - - - - 378

Liver, New Excretory Function, - - - - - - 142
Lupus, - - - - - - - - - - 429
Larrey, Anecdote of Baron, - - - - - - - 480
Logic, Medical—Armor, - - - - - - - - 563
Lead Pipe, Prevent. Injury from, - - - - - - 570

Military Hospitals—Lackey, - - - - 7, 94
Military Surgery, Gunshot Fractures, - - - - 339
Medicines, Cumulative—Fleming, - - - - 22
Miscellaneous, - - - - - - - 87
Morbid Anatomy, - - - - - - 184
Military Surgery, Resections, - - - - 212
Mormonism, Physiology of, - - - - - 307
Miller, DeLaskie, M. D., - - - - - - 323
Michigan University—The "Swamp Angel," - 324
Minor Surgery—Packard, - - - - 328
Mamma, Purulent Sinuses in, - - - 333
Mercury in Syphilis, - - - - - 333
Medicated Cigarettes, - - - - - - 457

Nostalgia, - - - - 83
Nature, Freak of—Eaton, - - - - - - 120
Necrosis, - - - - - - - 221
Nails, Ulcers, Sesquichloride of Iron, - - 400
Nerve, Great Sympathetic, - - - - 452
Neuroma of Optic Nerve, - - - - - 566

Ovary Prolapsus, . . . . . . . 117
Os Uteri Imperforate, . . . . . 121
Ovariotomy, . . . . . . . 217
Ovarian Dropsy, Iodine Injection, . . . . 218
Ophthalmic Hospital, N. Y., . . . . . 229
Obstetrics, Science and Art—Meigs, . . . . 233
Oxygen Gas, . . . . . . . . 382
Osteoscopic Pains, . . . . . . 430
Ovariotomy in France, . . . . . . 480
Ophthalmic Medicine—*Jones*, . . . . . . 561
Obituary—Geo. Hayward, . . . . . 569
do Chas. P. Marsh, M. D., . . . . . 573

Presentation, Shoulder—Eaton, . . . . . . 4
Podophyllin—Hoag, . . . . . . . 10
Psychical Diseases—Greeds, . . . . . 14
Pregnancy Inf. on Tuberculosis, . . . . 26
Paine's Institutes, . . . . . . . 39
People's Dental Journal, . . . . 44
Puerperal Convulsions, . . . . . . 74
Pertussis, . . . . . . . . 80, 221
Pneumonia, Clinical Lecture—Chambers, . . . 102
Painless Parturition, . . . . . . . 199
Pultaceous Exudations and False Membrane, . . . 203
Precocious Pregnancy and Parturition, . . . 216
Phthisis, . . . . . . . . . 223
Pyramidal Cataract—Holmes, . . . . 289

Parotid Gland, Extirpation of—Brainard, . . 337
Perjury by a Surgeon, . . . . . . 382
Practice, Rumored New Work, . . . . 383
Pharmacopœia, U. S., . . . . . . 426
Patients, A Word to, . . . . . 464
Pus, Physiological and Pathological, . . . 465
Pyrophosphate of Iron and Calisaya Bark, . . 527
Pancoast's Styptic, . . . . . . 474
Professional Acknowledgments, . . . . 475
Pyæmia or Leukæmia—Weeks, . . . 554
Phthisis, Open Air Treatment, . 575

Quinine, Administration of, . . . 221
do Therapeutic Action, . . . . . 432

Rush Medical College, Graduates, . . . 35
do do do Resolutions of Class, . . 48
do do do Valedictory—Rea, . . 50
do do do Progress and Condition, . 426, 526
Report Jacksonville Insane Asylum, . . . 45
Rheumatism, Clinical Lecture—Chambers, . . 154
Radius, Partial Dislocation of Head in Children, . 360
Rheumatism, Clinical Lecture—Fuller, . . 362
Reform School, . . . . . . . 427
Radius, Dislocation—Weller, . . . . . 436
Rigor, Undue in Management of Sick, . . . 459

Sanitary Aspects Recent Campaign, . . . 31
Surgeon, Eloquent Tribute to, . . . . . 47
Syphilis, Employment of Merc. and Iod. Pot., . 201
Scarlatina and Puerperal State, . . . . 210
Syphilis, . . . . . . . . 222
Sub Involution of Uterus after Parturition, . . 227
Skin, Diseases of—Erasmus Wilson, . . . 234
Surgical Cases after Battles—Clark, . . . 241
Strychnia as a Poison, . . . . . . 271
Surgeon-General's Orders, . . 254, 285, 310
Spina Bifida, . . . . . 320
Simpson, Diseases of Women and Children, . . 328
Surgery, English, . . 401
Syphilis Communicated by Vaccination, . . . 403
Scavengers of Nature, . . . . . 412
Societies Medical, Work for, . . . 422, 466
Stimulants in the Army, . . . . . 455
Stomach, Immunity from Digestion, . . . 519
Symptoms and Signs, . . . . . . 528
Sewage, . . . . . . . . 570

Typhus and Typhoid Fever, . . . . . 69
Trismus Nascentium, . . . . . . 72
Tissue Metamorphosis, Inf. of Salt and Coffee, . 204
Thoracentesis, . . . . . 217
Tooth Powder, . . . . . . . . 217
Transparent Paper, . . . . . . 236
Tetanus, . . . . . 429
Trephining Case, . . . . . . . 512

Uterus and Ovaries, Removal of, . . . . 334
Uterine Disease, Medicated Pessaries, . . . 407
Uterine Polypus, Removal, . . . . 419
Ulcers Indolent, Lotion, . . . . . 430

Varicose Veins, . . . . . . 218
Vaccination during Pregnancy, . . . . 232
Vade Mecum—Mendenhall, . . . . 327
Vertebrate Skeleton, . . . . . . 539

Wisconsin Institute for Blind, . 45
Water Oxygenated, . . . . . . 108
Wales, Prince of, Medical Officers, . . 220
Western Medical Department of Army, . . . 410
Warm Water Dressings, . 438
Women, Retreat for Intemperate, . 547

Yellow Fever, Phosphorus, . . 473

